# Maternities

**Published:** 1905-06

**Authors:** 


### Books and Magazines.

Maternities. A book concerning the care of the prospective mother and the child. By Chas. E. Paddock, M. D., Professor of Obstetrics, Chicago Post-Graduate Medical School; Assistant Professor of Obstetrics, Rush Medical College. Chicago: Cloyd J. Head & Co. 1905. Pp. 190; price, $1.25.

The aim of this book is to "aid the prospective mother during her pregnancy and to guide her in those trying days and weeks after the baby has come and the trained nurse has been dismissed."

It consists of Part I—the Mother, and gives the hygiene of the pregnant and puerperal state and much valuable advice; and Part II—the Baby, its cares, its toilet, its food, diseases and injuries. It is a valuable little work and it will be useful to those millions of mothers who have no "trained nurse" and too often no physician. I wish they all could read it.

